# Navigating How to Initiate Tenapanor Therapy in the Real World

**DOI:** 10.34067/KID.0000000000000488

**Published:** 2024-07-25

**Authors:** Keisuke Soeda, Hirotaka Komaba

**Affiliations:** 1 Division of Nephrology, Endocrinology and Metabolism, Tokai University School of Medicine, Isehara, Japan; 2 The Institute of Medical Sciences, Tokai University, Isehara, Japan

**Keywords:** hyperphosphatemia

Hyperphosphatemia is a common complication of kidney failure and is associated with vascular calcification and mortality. Strategies to control hyperphosphatemia include dietary phosphate restriction, the use of phosphate binders, and ensuring adequate dialysis.^1^ However, adequate control of serum phosphate levels remains a challenge because of the limited efficacy of phosphate binders, variable adherence to taking these pills, and the difficulty of dietary phosphate restriction. Tenapanor is a potential new option for the treatment of hyperphosphatemia. Unlike conventional phosphate binders, it directly inhibits intestinal phosphate absorption by selectively inhibiting sodium/hydrogen exchanger isoform 3, which most likely induces a conformational change in tight junction proteins leading to inhibition of paracellular phosphate transport.^2^

The efficacy of tenapanor in controlling hyperphosphatemia has been demonstrated in several clinical studies. Previous studies have shown that tenapanor monotherapy significantly reduced serum phosphate levels compared with placebo or sevelamer in dialysis patients.^3–5^ Subsequent studies have further demonstrated the efficacy of combination therapy with tenapanor and phosphate binders in controlling serum phosphate levels.^6,7^ In these studies, tenapanor was started at a dose of 30 mg twice daily, but subsequent studies in Asian dialysis patients using a protocol that gradually increased the dose from 5 mg twice daily also confirmed a significant benefit in phosphate control.^8^ Tenapanor is known to cause soft stools or diarrhea as a result of sodium/hydrogen exchanger isoform 3 inhibition but has shown an acceptable safety and tolerability profile in previous studies.^3–8^

In the May issue of *Kidney360*, Sprague and colleagues report the results of the OPTIMIZE study,^9^ which used three different initiation strategies for tenapanor. The primary target population was dialysis patients with serum phosphate levels >5.5 mg/dl despite the use of conventional phosphate binders. Participants were randomized to either a complete switch from phosphate binders to tenapanor (straight switch group) or to a regimen in which the phosphate binder dosage was reduced by more than 50% with the addition of tenapanor (binder reduction group). The study also included dialysis patients who were not receiving phosphate binders and had phosphate levels >4.5 mg/dl, in whom tenapanor was initiated as monotherapy (binder-naive group). In all groups, participants received 30 mg of tenapanor twice daily for 10 weeks, followed by an optional 16-week open-label extension.

Over the course of the study, there was a different pattern of the longitudinal changes in serum phosphate levels between the binder reduction and straight switch groups. In the binder reduction group, there was a large decrease in serum phosphate levels 1 week after starting tenapanor, and the low levels were maintained until week 9, after which there was an increasing trend. In the straight switch group, the initial decrease in phosphate levels after starting tenapanor was relatively small, but there was a gradual decrease in serum phosphate levels throughout the period. As a result, serum phosphate levels at the end of week 10 were comparable, with a mean level of 6.0–6.5 mg/dl in both groups. In the binder-naive group, serum phosphate levels decreased significantly immediately after starting tenapanor, and the mean serum phosphate level at the end of week 10 was 5.0–5.5 mg/dl, with more than 60% of patients achieving phosphate levels ≤5.5 mg/dl. As expected, the most common adverse event was diarrhea, but the incidence was approximately 40%, which is lower than in previous studies, and few patients discontinued tenapanor because of diarrhea.

Phosphate binders are widely used as the only currently available drugs to control hyperphosphatemia, and an important question is how to use tenapanor in combination with or without phosphate binders. A unique feature of the OPTIMIZE study^9^ was that it evaluated three different strategies for initiating tenapanor, each of which appears to be appropriate for real-world clinical practice. Importantly, the study protocol allowed the dose of the phosphate binder to be reduced as serum phosphate levels declined while maintaining the dose of tenapanor at 30 mg twice daily, in contrast to the AMPLIFY study,^6^ which reduced tenapanor at low phosphate levels while maintaining the dose of the phosphate binder. Thus, it could be said that the OPTIMIZE study positioned tenapanor, rather than phosphate binders, as the mainstay of treatment for hyperphosphatemia.

There are several possible explanations for the notable differences in longitudinal changes in serum phosphate between the straight switch and binder reduction groups. In the straight switch group, serum phosphate levels decreased relatively little, presumably because phosphate binders were discontinued with the initiation of tenapanor, but there was a slow but steady decrease in serum phosphate levels with the resumption and gradual increase of phosphate binders. In the binder reduction group, serum phosphate levels decreased more rapidly, presumably because the effect of initiating tenapanor exceeded that of reducing phosphate binders by half. The reason for the subsequent gradual increase in phosphate levels is unknown, but given the continued administration of tenapanor and phosphate binders, this may be explained by a decrease in adherence or a relaxation of the dietary phosphate restriction.

The comparable efficacy at the end of the study between the straight switch and binder reduction groups has implications for the use of tenapanor in clinical practice. The results suggest that the choice between the two approaches should be individualized based on patient preference and phosphate control. For example, for patients with uncontrolled hyperphosphatemia and a high pill burden or poor adherence, it may be reasonable to choose the straight switch approach and advise the patient to be sure to take tenapanor, with resumption of phosphate binders as needed. On the other hand, for patients with good adherence but inadequate control of hyperphosphatemia, it may be reasonable to choose the binder reduction approach or to add tenapanor without reducing phosphate binders. In addition, although not tested in this study, the use of tenapanor may improve patient satisfaction and nutritional status even in patients with well-controlled hyperphosphatemia by reducing pill burden and relaxing dietary phosphate restrictions. The use of tenapanor may also be considered in patients with vascular calcification, as a recent study demonstrated the benefit of intensive phosphate control in attenuating the progression of vascular calcification.^10^ In the future, individualizing the use of tenapanor appears to be an important step in managing phosphate levels in clinical practice.

Because patient initiative is critical to controlling serum phosphate levels, patient preference for phosphate-lowering treatment is a very important issue. In this study,^9^ the majority of participants in the straight switch and binder reduction groups responded to the patient experience questionnaires, with more than 80% in both groups reporting an improvement in their phosphate management routine. In addition, approximately 70% reported that their phosphate management was much less or somewhat less difficult. Given that patient preferences are likely to influence medication adherence and treatment efficacy, these data are meaningful and support the practical value of tenapanor.

The results in the binder-naive group are also important because it was the first to evaluate the efficacy of tenapanor in patients not treated with phosphate binders. Similar to previous studies, tenapanor was shown to be effective in this population, supporting its potential as a first-line treatment. However, the choice between tenapanor and phosphate binders as initial treatment requires further consideration. If the cost of tenapanor is higher than that of existing phosphate binders, as is the case in Japan, starting with phosphate binders would make more sense from a health economic perspective.

In terms of safety, the incidence of diarrhea was approximately 40%, which was lower than in previous studies, and few patients discontinued tenapanor because of diarrhea.^9^ As the authors also suggest, it is likely that pretrial education about the side effects of tenapanor helped reduce the incidence of diarrhea and drug discontinuation. These findings support the notion that education before the use of tenapanor is important to reduce patient anxiety, maintain adherence, and maximize efficacy in real-world clinical practice. It is also interesting to note that that the incidence of adverse events was lower in the binder-naive group. It would be worthwhile to investigate whether and how concurrent use of phosphate binders affects the incidence or severity of diarrhea associated with tenapanor.

Finally, while this study confirmed the efficacy of tenapanor, it should be noted that only 30%–40% of patients in the binder reduction and straight switch groups achieved serum phosphate levels of ≤5.5 mg/dl at the end of the study.^9^ This finding was similar to that observed in the AMPLIFY study,^6^ in which tenapanor was added to phosphate binder therapy. These results underscore the challenge of controlling serum phosphate levels, even with the use of tenapanor, in patients with uncontrolled hyperphosphatemia who often have high dietary phosphate intake and poor medication adherence. Further efforts are needed to establish a more optimized approach to improve phosphate management in treatment-resistant populations.

In conclusion, this study evaluated three different approaches to tenapanor initiation and provided important evidence for its clinical application by demonstrating the efficacy of multiple ways to use tenapanor. To further establish the clinical value of tenapanor, the next critical step will be testing the efficacy in the real-world setting.
